# Synovial Lipomatosis in the Knee Joint: A Report of a Rare Condition

**DOI:** 10.7759/cureus.71391

**Published:** 2024-10-13

**Authors:** Anteshwar Birajdar, Vikram Reddy, Rajeev Reddy

**Affiliations:** 1 Orthopedics, Dr. D.Y. Patil Medical College, Hospital and Research Centre, Pune, IND

**Keywords:** arthroscopic debridement, diagnostic arthroscopy, knee swelling, lipoma arborescens, synovial lipomatosis

## Abstract

Synovial lipomatosis is an uncommon, benign intra-articular lesion that resembles a lipoma and typically affects the synovial lining of the knee joint. It can cause swelling and pain in the joint, as well as restrict movement. Mature fat, proliferating and infiltrating the hypertrophic synovial villi, primarily causes the lesion. Although the hip, glenohumeral, wrist, and ankle joints have also been reported to be affected, the knee joint is a common site of the condition. This benign condition has an unknown cause. This case report describes a 47-year-old male with right knee pain, swelling, and mechanical symptoms. Treatment options include conservative or surgical treatment, depending on the patient's symptom severity. In this case, we opted for surgical management, which included arthroscopic debridement and synovectomy. The occurrence of synovial lipomatosis in the knee is rare, so the treating surgeon must maintain a high level of suspicion.

## Introduction

Synovial lipomatosis is an uncommon benign lesion that typically affects the suprapatellar pouch of the knee [1]. This benign development of sub-synovial adipose tissue is also known as lipoma arborescens. The cause is unknown, although it is more likely reactive than neoplastic. The word arbor (tree in Latin) refers to the distinctive frond-like and villous synovial extensions formed by the resultant fatty mass, which lead to a macroscopic appearance like a leafy tree. Synovial lipomatosis appears to develop at any age. Males in their middle years account for the majority of reported instances. Patients frequently report swelling and pain in the affected joint. Synovial lipomatosis has been known to occur in the wrist and ankle, though commonly it affects the knee [2]. The recommended treatment is a full synovectomy, which can be performed via formal open debridement or arthroscopic techniques. Although the most effective approach is arthroscopic synovectomy and debridement, both have presented favorable results despite a lack of follow-up outcome data [3]. While the site of symptoms varies, histomorphology and radiologic examination appear more constant. This case report describes the presentation, key diagnostic features, and treatment plan for this unusual entity.

## Case presentation

**Figure 1:**
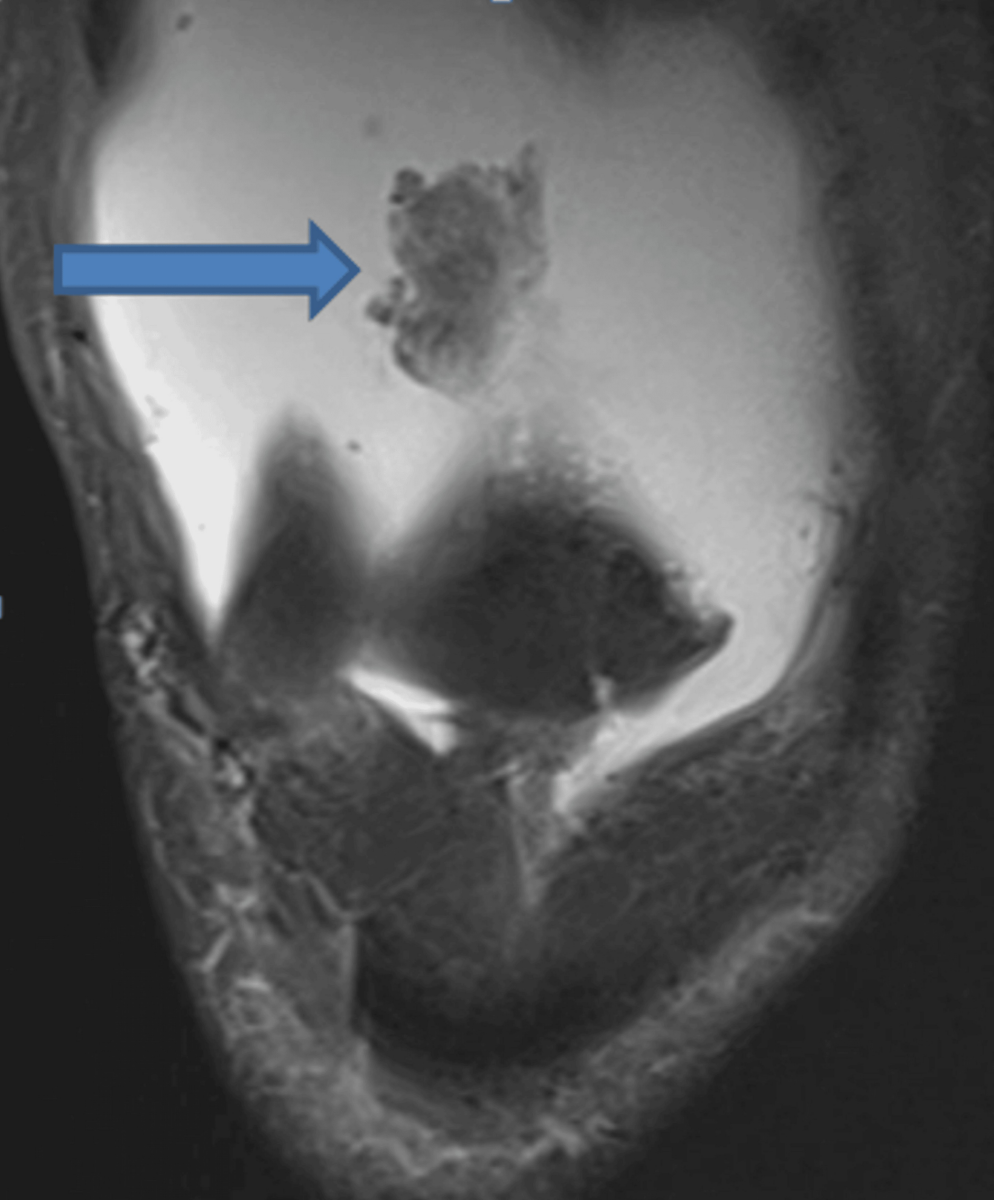
Coronal section of right knee MRI demonstrating leaf-like projections of tissue arising from the suprapatellar pouch, which has the same signal intensity as fat.

**Figure 2:**
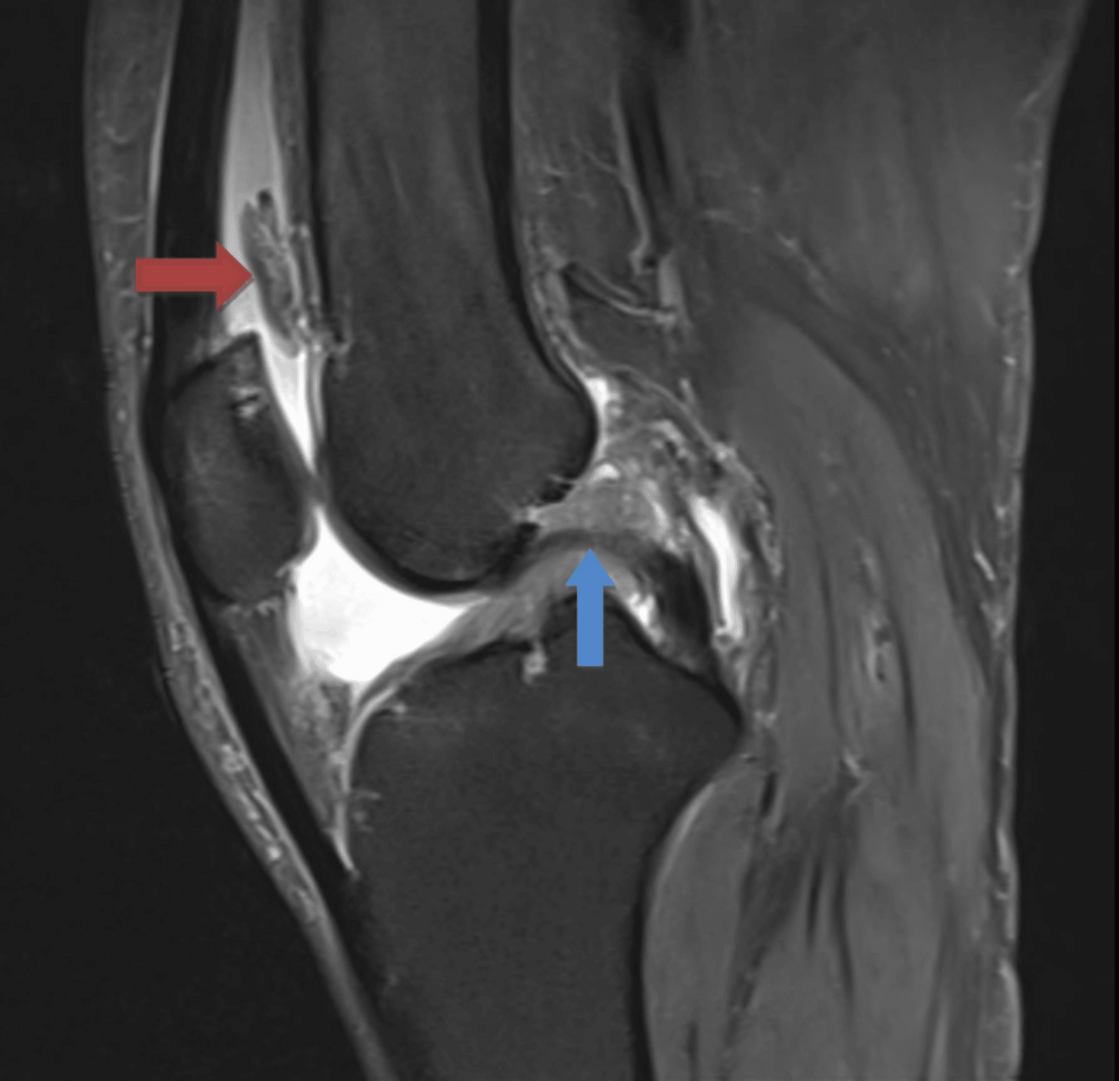
Sagittal section of the MRI of the right knee Red arrow: Frond-like mass arising from the suprapatellar pouch, Blue arrow: Mucoid degeneration of the ACL ACL: Anterior cruciate ligament

**Figure 3:**
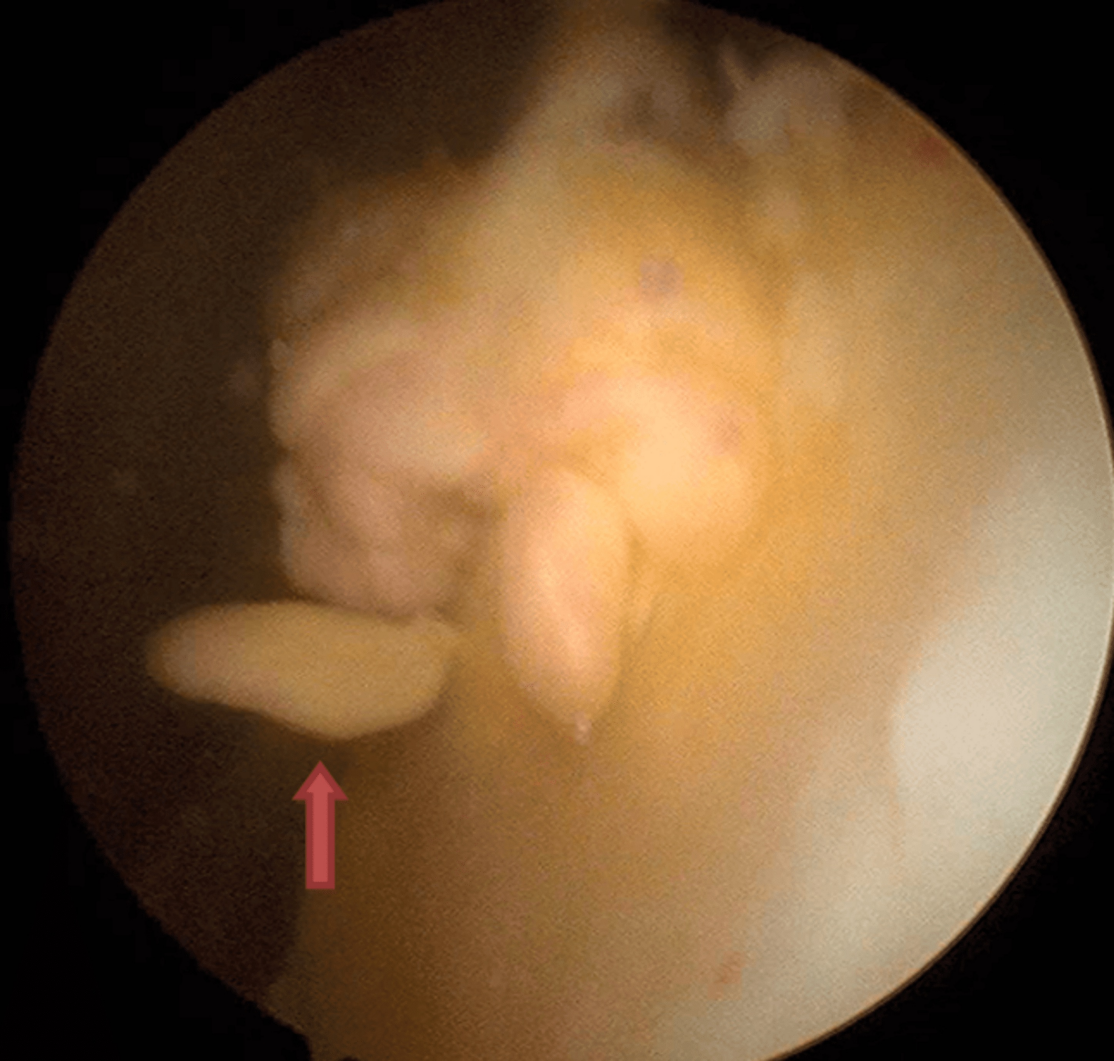
Arthroscopic image of the suprapatellar pouch The red arrow points at the hypertrophic synovial villi.

A 47-year-old male presented to the outpatient clinic with swelling and mild pain in his right knee for five years that had gotten worse over the previous two months. The pain worsened with activity and was relieved by taking rest. He had no mechanical symptoms like locking and had no recent weight loss, anorexia, fever, or nocturnal cramps. He had no other comorbidities and no other complaints. On inspection, the patient appeared to be well-fed, afebrile, and healthy. The right knee swelling was on the anteromedial aspect, non-tender, without warmth, with a range of motion of 0° to 120° degrees, no ligament laxity, and no distal neurovascular impairment. The swelling was generally limited to the suprapatellar area and not associated with the underlying anatomy, bone, or skin that covers it. The clinical symptoms led to the differential diagnosis of ganglion cyst, synovial lipomatosis, extruded meniscus, pigmented villonodular synovitis, liposarcoma, and synovial hemangioma alterations. A plain radiograph of the knee revealed a significant reduction in medial joint space. An MRI showed hypertrophic synovium and mild effusion in the suprapatellar fossa. It revealed mucoid degeneration of the anterior cruciate ligament (ACL) and villous-like projections mainly from the suprapatellar pouch that has the same signal intensity as fat, suggesting synovial lipomatosis (Figures 1-2). The patient underwent arthroscopic synovectomy and debridement and was sent for a biopsy (Figure 3).

**Figure 4:**
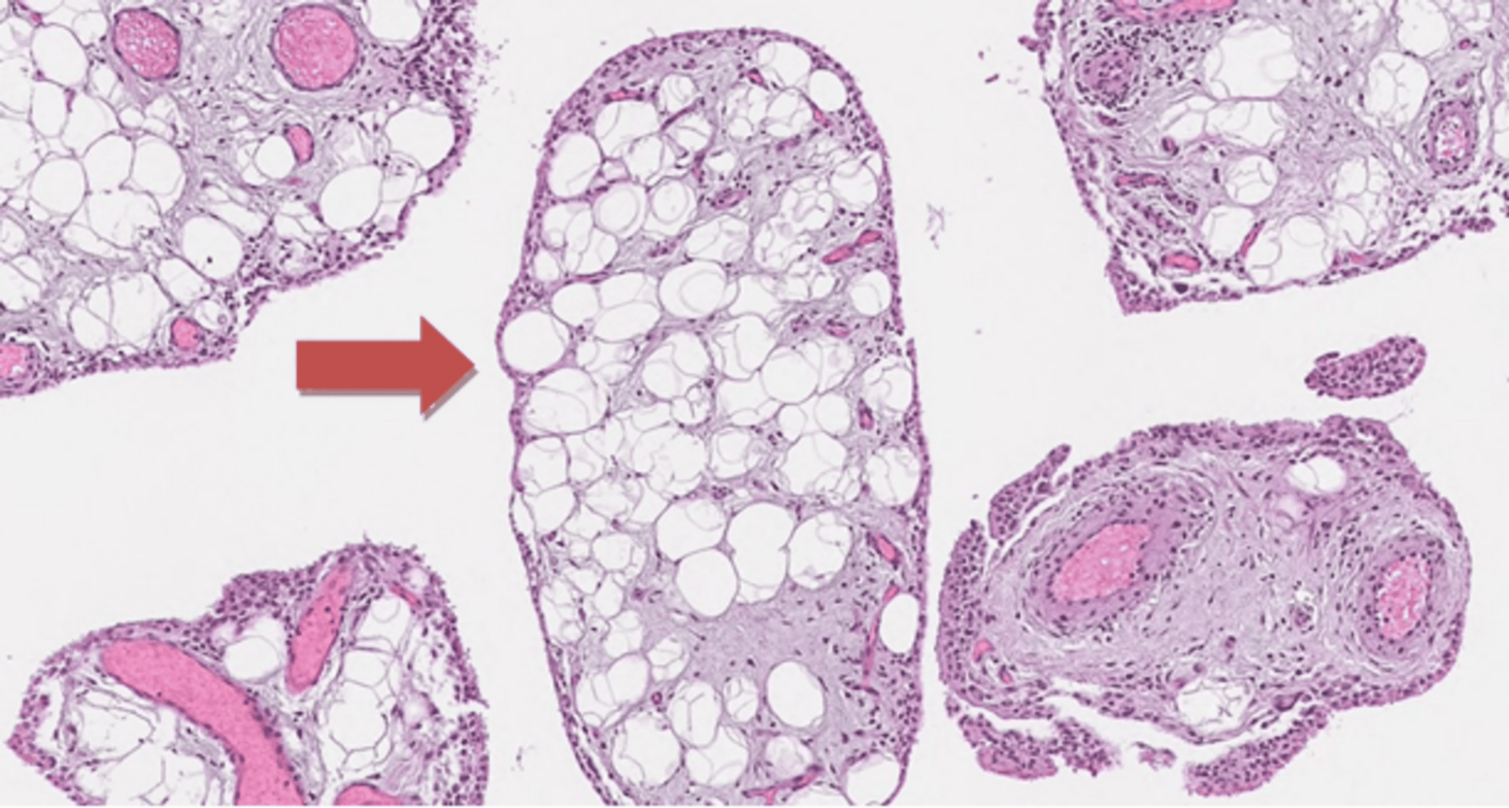
Histological examination of the excised tissue shows synovial hyperplasia with mature fat cells extending into the synovial lining. The red arrow points at the mature fat cells beneath the synovial lining in hematoxylin and eosin (H&E) staining (100x).

Histopathological examination revealed mature adipose tissue cells, red cell extravasation, and papillary projections with a synovial epithelial lining, confirming the diagnosis of synovial lipomatosis (Figure 4). Debulking of the mucoid anterior cruciate ligament was done. The patient was initially immobilized in a long knee brace, after which the patient was mobilized according to the ACL rehabilitation protocol. 

## Discussion

Pain and swelling in the joints are the hallmarks of synovial lipomatosis [4]. In 1904, German doctor Albert Hoffa gave the first report on synovial lipomatosis at the American Medical Association's annual meeting in New Jersey [5]. The precise etiology of this illness remains unknown. This condition is not a tumor but rather a nonspecific reactive proliferation of the synovial membrane brought on by rheumatoid arthritis, chronic synovitis, or joint trauma [6]. The knee is the joint that is most commonly affected by this condition, though it has been reported in several other joints as well [6,7].

Synovial lipomatosis can be distinguished from other diseases by a few specific features. Distinguishing synovial lipomatosis from pigmented villonodular synovitis is possible based on hemosiderin and bleeding-free histology [8]. With MRI, synovial chondromatosis can be differentiated from synovial lipomatosis, even though radiographs usually show a clear diagnosis. This is due to the characteristic chondroid calcifications and ossified loose bodies that exhibit typical features of higher signal marrow fat centrally and low signal cortical bone peripherally [9]. Differential diagnoses include other benign synovial lesions containing fat based on histopathology, such as synovial lipoma and Hoffa disease. Hoffa's disease is caused by the infrapatellar fat pad compressing against the patella and femoral condyle. Under a microscope, mature fat cells gradually replace the subsynovial layer in synovial lipomatosis, and there is a slight infiltration of mononuclear inflammatory cells. Even though they are covered in synovium, intra-articular lipomas do not originate from or replace the subsynovial layer. Furthermore, villous fronds and intra-articular lipomas are not similar [10]. Synovial lipomatosis can be treated with both open and arthroscopic synovectomy techniques that have a relatively shorter recovery period. For this reason, arthroscopic debridement and synovectomy are preferred. Recurrences are uncommon.

## Conclusions

Even though synovial lipomatosis usually develops spontaneously, it could be linked to a degenerative process. It could also develop as a secondary process after a long-term joint condition such as osteoarthritis. Our case study raises awareness of this rare illness. If the surgeon does not have a high index of suspicion, this entity might be overlooked, particularly when the patient is young and has chronic refractory knee pain with an otherwise negative workup. It is advised to consider arthroscopic synovectomy and debridement if MRI results support a diagnosis of synovial lipomatosis. With arthroscopic debridement, patients will have a shorter recovery period than open procedures. Even though our case study only includes one person, it shows why more research is necessary because this is a rare, distinct entity with effective treatment options.
